# Atezolizumab-Induced Generalized Cutaneous Lichen Planus in a Patient With Metastatic Small Cell Lung Cancer Successfully Treated With Isotretinoin: A Case Report

**DOI:** 10.7759/cureus.77579

**Published:** 2025-01-17

**Authors:** Cătălina-Ioana Naum, Beatrice Bălăceanu-Gurău, Alexandra Timofte, Irina Tudose, Mara M Mihai

**Affiliations:** 1 Dermatology Department, Elias Emergency University Hospital, Bucharest, ROU; 2 Oncologic Dermatology Department, Carol Davila University of Medicine and Pharmacy, Bucharest, ROU; 3 Pathology Department, Elias Emergency University Hospital, Bucharest, ROU

**Keywords:** atezolizumab, generalized lichen planus, immune checkpoint inhibitors, isotretinoin, small cell lung cancer

## Abstract

Immune checkpoint inhibitors, now widely used in treating various malignancies, increase the risk of autoimmune reactions and immune-related adverse events (irAEs), with skin toxicities being the most frequent. These agents enhance the immune response against tumors by blocking the suppression of cytotoxic T lymphocytes.

Here, we report a rare case of generalized cutaneous lichen planus induced by atezolizumab, an immune checkpoint inhibitor administered for small cell lung cancer. After consulting with the oncologist, we opted to initiate isotretinoin as the first-line treatment. Considering the patient's oncologic status and multiple comorbidities, we aimed to avoid systemic corticosteroids due to their potential side effects.

This case was effectively managed with low-dose oral isotretinoin alongside high-potency topical corticosteroids. It emphasizes the need to consider retinoids as a potential treatment option for various dermatological conditions beyond acne. Isotretinoin may be beneficial in treating lichen planus by influencing cellular proliferation and promoting epithelial differentiation, though its exact mechanism remains uncertain. Additionally, it has notable anti-inflammatory effects and modulates immune responses. In such cases, isotretinoin may also enhance the therapeutic effects of topical corticosteroids through a synergistic interaction.

## Introduction

Immune checkpoint inhibitors targeting programmed cell death protein-1 (PD-1) and programmed death ligand-1 (PD-L1) are increasingly utilized in the treatment of various malignancies, including melanoma, non-small cell lung cancer, small cell lung cancer, liver cancer, alveolar soft part sarcoma, and hematologic cancers. These agents work by preventing the suppression of cytotoxic T lymphocytes, thereby enhancing the immune response against tumors. However, they also raise the risk of autoimmunity and immune-related adverse events (irAEs), with cutaneous effects being the most common. Reported dermatologic side effects include a range of eruptions (maculopapular, lichenoid, psoriasiform, vesiculo-bullous), vitiligo, prurigo, and others [1]. Multiple studies highlight the predictive significance of these skin eruptions and their subtypes concerning cancer prognosis.

The incidence of cutaneous irAEs in patients receiving immune checkpoint inhibitors ranges from 30% to 60% [1].

The pathogenesis of cutaneous irAEs remains unclear, but several hypotheses have been proposed. These include the activation of T cells targeting shared antigens in both normal tissues and tumor cells, the increased release of pro-inflammatory cytokines leading to immune responses in specific tissues or organs, a potential link between certain human leukocyte antigen (HLA) variants and organ-specific irAEs, and the exacerbation of drug eruptions induced by concurrent medications [1].

In this report, we present an unusual case of generalized cutaneous lichen planus caused by atezolizumab, an immune checkpoint inhibitor used in small cell lung cancer treatment.

This case report was previously presented as a conference poster at the 33rd Congress of the European Academy of Dermatology and Venereology, Amsterdam, on September 25-28, 2024.

## Case presentation

**Figure 1:**
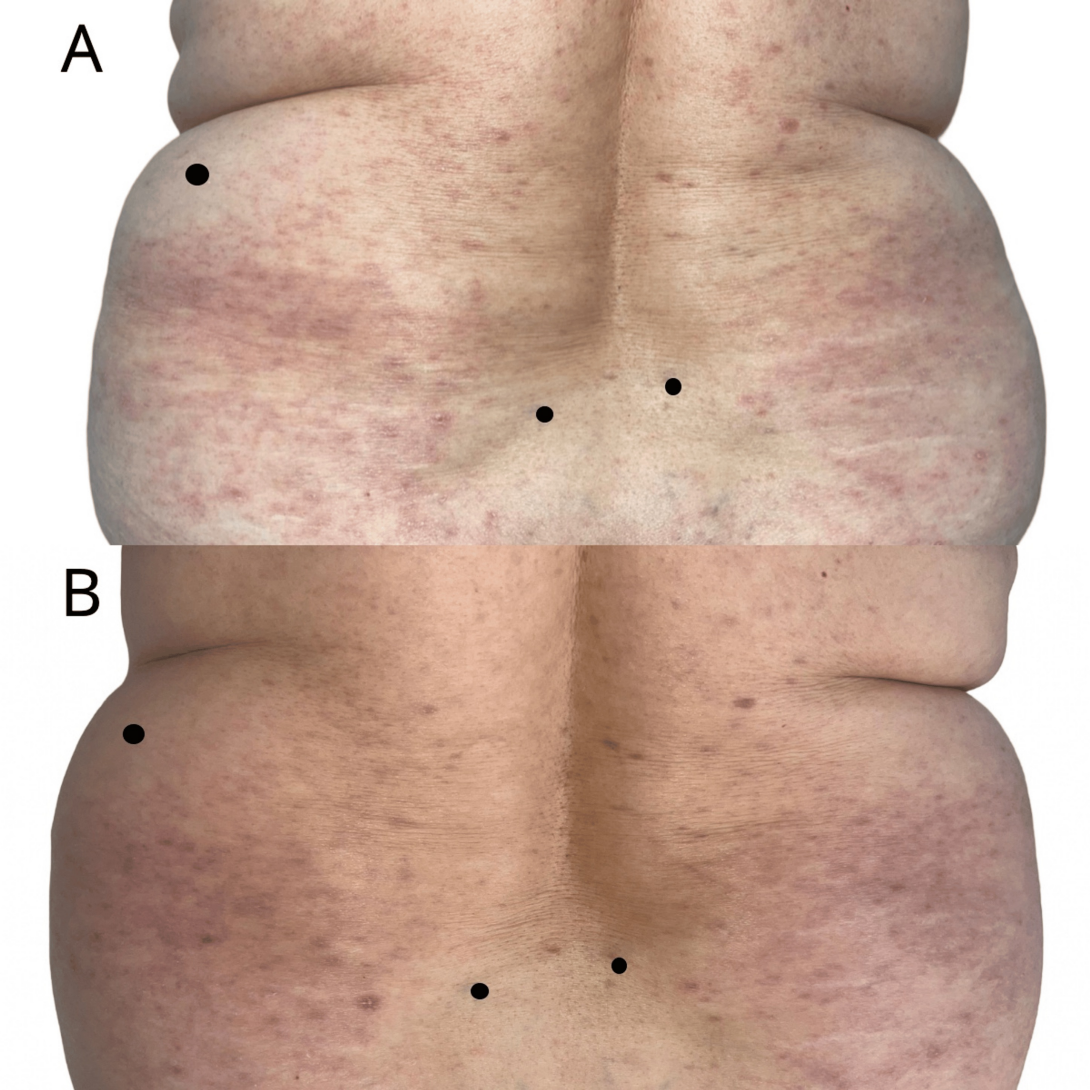
Clinical findings at initial presentation (A) and after one month of treatment (B): pruritic eruption characterized by shiny, purpuric, polygonal papules ranging from 4-5 mm to 1 cm in size, coalescing into plaques, located on the posterior trunk, showing favorable response to treatment

**Figure 2:**
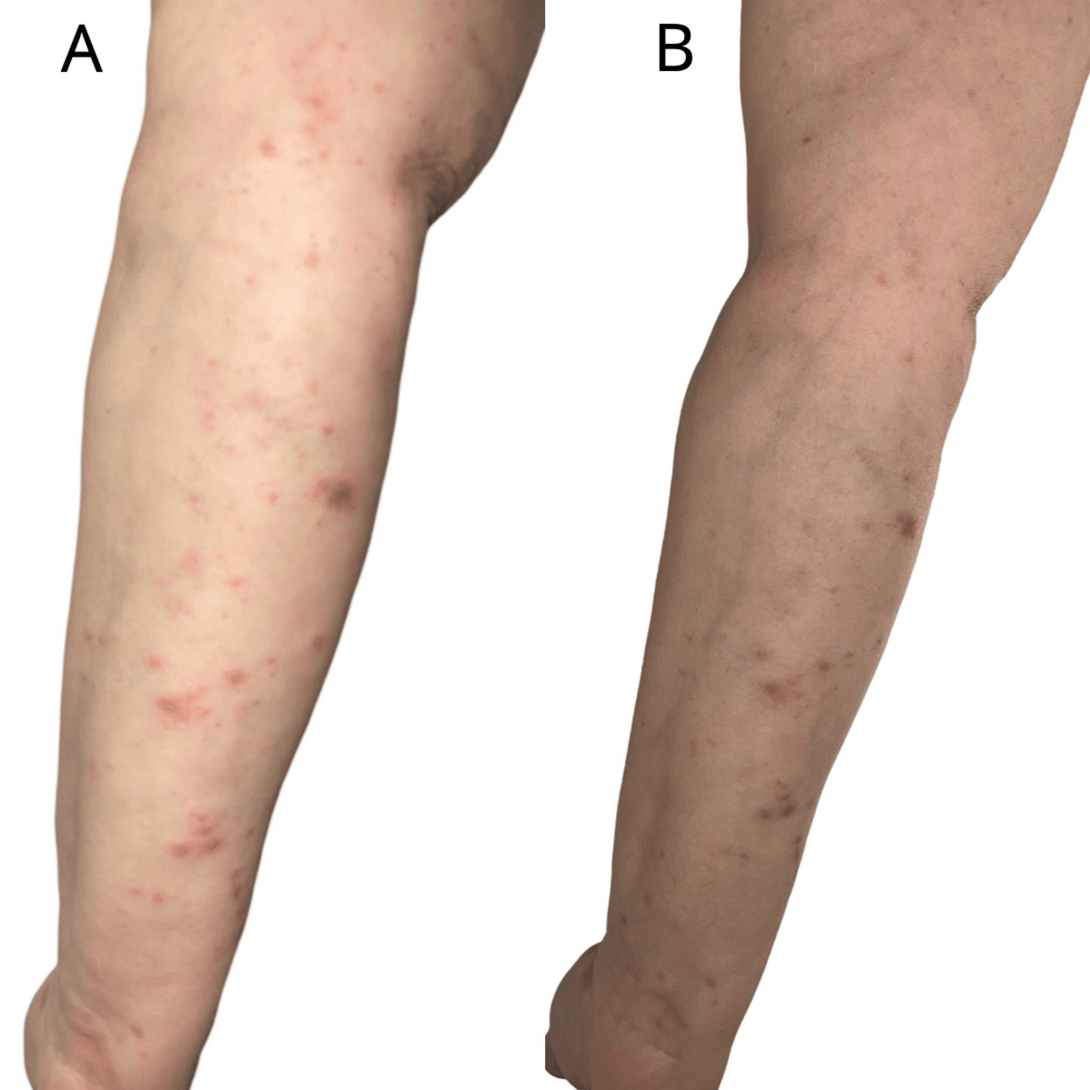
Clinical findings at initial presentation (A) and after one month of treatment (B): pruritic eruption characterized by shiny, purpuric, polygonal papules, located on the left upper limb, showing favorable response to treatment

**Figure 3:**
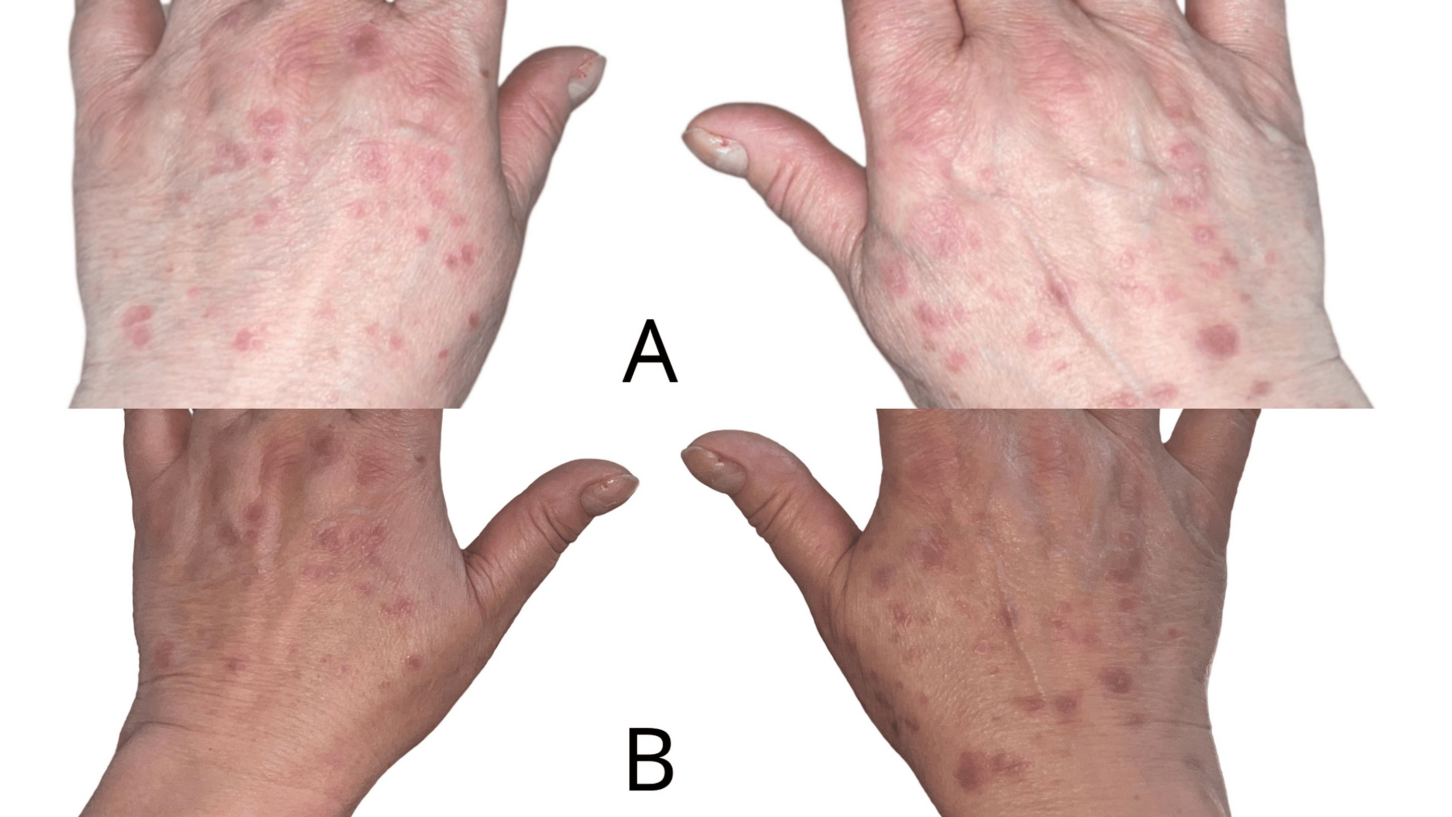
Clinical findings at initial presentation (A) and after one month of treatment (B): pruritic eruption characterized by shiny, purpuric, polygonal papules, located on the backs of the hands, showing favorable response to treatment

We present the case of a 59-year-old female patient with a history of stage IVA (T4N2M1a) small cell lung cancer, affecting the right lower lobe, complicated by carcinomatous lymphangitis, pleural and pericardial effusion, and supradiaphragmatic adenopathies. Despite progression under chemotherapy and radiotherapy, the patient was being treated with atezolizumab, an anti-PD-L1 immune checkpoint inhibitor. She presented to our department with a generalized pruritic eruption characterized by shiny, purpuric, polygonal papules ranging from 4-5 mm to 1 cm in size, coalescing into plaques (Figures 1-3). The lesions predominantly affected the trunk and extremities and showed no improvement with high-potency topical corticosteroids.

**Figure 4:**
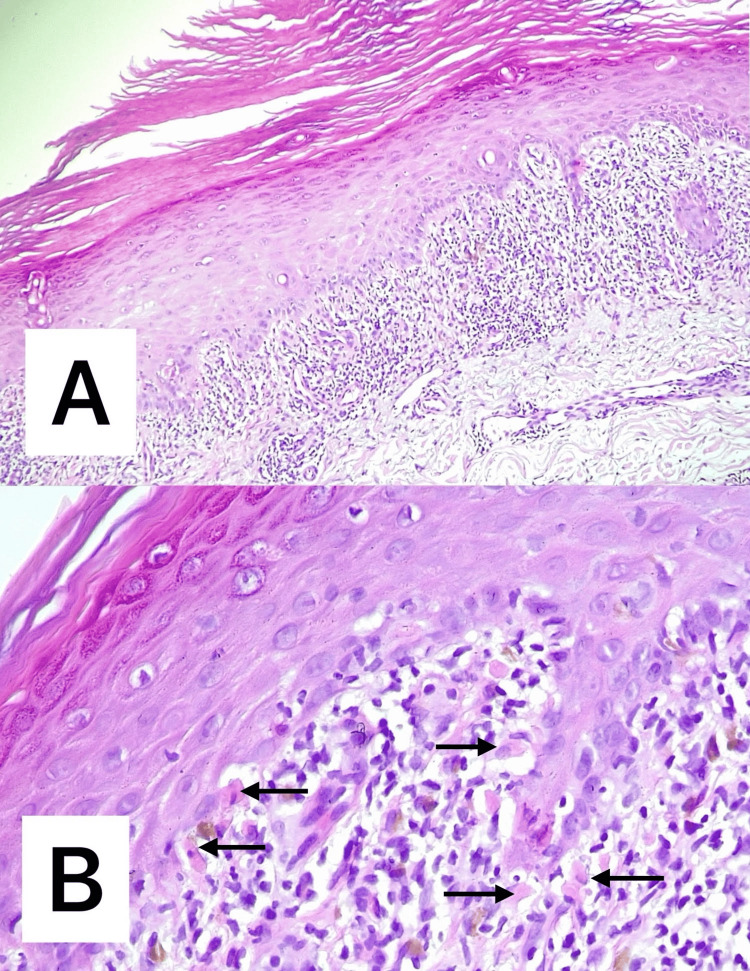
Histopathological findings: (A) moderate focal orthokeratosis, hypergranulosis, and acanthosis and (B) rare apoptotic keratinocytes (Civatte bodies (arrows)) at the basal layer, accompanied by a moderate band-like lymphocytic infiltrate at the dermo-epidermal junction and vacuolar degeneration of the basal epithelium (A: H&E ×100; B: H&E ×200) H&E: hematoxylin and eosin

Laboratory tests revealed leukopenia of 2.62×10³/μl (reference range: 4.6-10.2×10³/μl), but no systemic viral infections were identified, and no other medications known to cause lichenoid reactions were involved. Histopathological analysis showed moderate focal orthokeratosis, hypergranulosis, and acanthosis, along with rare apoptotic keratinocytes (Civatte bodies) at the basal layer, accompanied by a moderate band-like lymphocytic infiltrate at the dermo-epidermal junction and vacuolar degeneration of the basal epithelium (Figure 4).

The patient was diagnosed with generalized lichen planus induced by atezolizumab for advanced small cell lung cancer. Given the extensive nature of the lesions and their significant impact on her quality of life, and with approval from the oncologist, we initiated low-dose isotretinoin (10 mg/day) for three months in combination with high-potency topical corticosteroids (0.1% methylprednisolone aceponate cream, two times a day). After one month of treatment, the patient's skin lesions showed marked improvement, with good clinical and laboratory tolerance (complete blood count, liver function tests, lipid panel), allowing continued atezolizumab therapy without interruption. 

## Discussion

The American Society of Clinical Oncology has developed a grading system to assess the severity of cutaneous irAEs. This system offers guidelines for managing these reactions, based on the extent of body surface area (BSA) involvement and associated symptoms. Cutaneous irAEs are categorized according to both clinical and histologic severity, as well as the percentage of BSA affected (Table 1) [1]. Based on the grading system, the patient can be classified as having grade 3 cutaneous irAEs, as more than 30% of the BSA is affected, with the presence of papules and associated symptoms. Grade 3 toxicities typically require pausing immune checkpoint inhibitors therapy and starting high-dose corticosteroids, with a gradual tapering over 4-6 weeks. In certain resistant cases, additional immunosuppressive treatments may be necessary [2]. Although the skin damage is extensive, it does not involve internal organ injury. Thus, high-dose systemic corticosteroid therapy is not a viable option given the risk-benefit balance. Additionally, considering the potential for prolonged progression of the skin condition, an effective treatment with a lower risk of toxicity from long-term use is preferred.

**Table 1:** Grading system for evaluating the severity of cutaneous irAEs BSA: body surface area; SJS: Stevens-Johnson syndrome; TEN: toxic epidermal necrolysis; irAEs: immune-related adverse events

Grade	Characteristic conditions
1	Asymptomatic+macules/papules <10% BSA
2	Macules/papules 10-30%±symptoms
3	Macules/papules >30%±symptoms
4	>30% BSA which represents the most severe skin reaction and can be life-threatening, including conditions like SJS, TEN, and bullous dermatitis. Careful management in an intensive care setting is essential

Notably, in contrast to spontaneous lichen planus, lichen planus-like eruptions induced by immune checkpoint inhibitors are rarely seen in the oral mucosa [1]. In the present case, the patient exhibited no lesions on the oral mucosa.

In terms of histopathological findings, in contrast to typical lichen planus, immune checkpoint inhibitors-induced lichen planus-like eruptions often display features such as epidermal spongiosis, parakeratosis, eosinophils, and necrosis [1]. 

Patients who experience irAEs induced by immune checkpoint inhibitors are typically thought to have a robust anti-tumor response. Spongiform changes and lichenoid reactions are linked to improved progression-free survival and reduced mortality, whereas vacuolar degeneration and superficial perivascular dermatitis are associated with a higher mortality risk [1].

Cutaneous lichen planus usually resolves spontaneously within one to two years, so treatment primarily focuses on alleviating pruritus and accelerating resolution by the use of superpotent or potent topical corticosteroids [3]. For more hypertrophic lichen planus, intralesional corticosteroids may be employed [3]. For widespread eruptions, other first-line treatments include slowly tapered oral corticosteroids, intramuscular injections of triamcinolone, oral retinoids such as acitretin or isotretinoin, as well as oral cyclosporine [3]. If there is no improvement, second-line therapies should be considered. These may include phototherapy with broadband or narrowband UVB, a combination of UV and acitretin, topical calcineurin inhibitors (tacrolimus, pimecrolimus), or sulfasalazine [3]. Third-line treatment options may also be considered: topical calcipotriol ointment; metronidazole; trimethoprim-sulfamethoxazole; hydroxychloroquine sulfate; antifungals like itraconazole, terbinafine, and griseofulvin; tetracycline; doxycycline; mycophenolate mofetil; azathioprine; methotrexate; cyclophosphamide; thalidomide; apremilast; adalimumab; ustekinumab; interferon a2b; alitretinoin; low-molecular-weight heparin; photodynamic therapy; extracorporeal photochemotherapy; neodymium-doped yttrium aluminum garnet (Nd:YAG) laser; or low-dose 308 nm excimer laser [3]. Kazemi et al. describe the swift resolution of lichen planus skin lesions and associated itching after treatment with dupilumab [4]. We decided to start isotretinoin therapy as the first-line treatment after consulting with the oncologist. Given that he was an oncologic patient with multiple comorbidities, we wanted to avoid initiating systemic corticosteroids due to their associated side effects.

Since its approval in 1982, isotretinoin has been the gold standard for treating severe acne. However, its anti-inflammatory properties suggest potential applicability as a versatile treatment option for a broader range of dermatologic conditions. Isotretinoin may effectively treat lichen planus by influencing cellular proliferation and keratinocyte differentiation, although the precise mechanism of action remains unclear [5]. Furthermore, isotretinoin exhibits significant anti-inflammatory properties and modulates immune responses. In these cases, it may also amplify the therapeutic effects of dermocorticoids through a synergistic effect. In clinical practice, the dosing and duration of isotretinoin therapy for dermatologic conditions vary between 0.1 and 8.2 mg/kg/day. Inflammatory conditions are typically managed with lower oral isotretinoin doses, ranging from 0.3 to 1 mg/kg/day [5]. Alghamdi et al. demonstrate the efficacy and safety of a treatment regimen for patients with unilateral linear lichen planus pigmentosus (LPP), revealing moderate improvement through low-dose isotretinoin in combination with topical therapy [6]. Additionally, isotretinoin was also recommended in the management of other types of skin rashes induced by oncologic medications, such as epidermal growth factor inhibitors [7], BRAF inhibitors, MEK inhibitors, and mTOR inhibitors [8]. In this case, the patient was treated with low-dose oral isotretinoin, and after one month, her skin lesions showed significant improvement, with favorable clinical response and laboratory tolerance. Acitretin, an alternative systemic retinoid for treating generalized lichen planus, was not recommended due to its unavailability in the country.

## Conclusions

Timely identification, accurate diagnosis, and early intervention for immunotherapy-induced dermatologic adverse effects are crucial to minimizing the risk of interrupting cancer treatment. This can be particularly challenging in the case of rare adverse events, such as generalized lichen planus induced by atezolizumab. Therefore, close interdisciplinary collaboration between dermatologists specializing in cutaneous reactions to oncologic therapies, dermatopathologists, and oncologists is essential for the prompt recognition and effective management of these conditions.

Although isotretinoin is considered a first-line therapy in lichen planus, it is rarely recommended in oncologic patients with dermatologic adverse reactions. To our knowledge, this is the first reported case of generalized lichen planus secondary to atezolizumab treated with isotretinoin. This highlights its potential beyond traditional acne treatment, showcasing its effectiveness as an anti-inflammatory and immunomodulatory agent in complex dermatologic conditions. Given the favorable response in this patient, isotretinoin may be a viable alternative or adjunct therapy, especially when systemic corticosteroids carry additional risks. This case report reinforces the value of isotretinoin's role in dermatology, suggesting its expanded use in managing select immune-related adverse effects and emphasizing the need for further research into its mechanisms and benefits in oncologic patients.
